# LncRNA FAM13A-AS1 Regulates Proliferation and Apoptosis of Cervical Cancer Cells by Targeting miRNA-205-3p/DDI2 Axis

**DOI:** 10.1155/2022/8411919

**Published:** 2022-06-23

**Authors:** Zhiqin Qiu, Lin He, Feng Yu, Hui Lv, Ye Zhou

**Affiliations:** Department of Obstetrics and Gynecology, Wuxi No. 2 People's Hospital, Affiliated Wuxi Clinical College of Nantong University, Wuxi, Jiangsu 214002, China

## Abstract

The aim of this study was to explore the function of long noncoding RNA (lncRNA) FAM13A-AS1 and its associated mechanism in cervical cancer. A total of 30 cervical cancer tissues and adjacent tissues were collected. Cervical cancer cell lines, including SiHa and HeLa, were transfected with constructs expressing LV-FAM13A-AS1, silencing RNA LV-siFAM13A-AS1, miRNA mimics, and miRNA inhibitors. RT-qPCR was used to detect the expression of FAM13A-AS1 in cervical cancer tissues, including SiHa, HeLa, and HUCEC cells. MTT, flow cytometry, and transwell assays were performed to explore the influence of FAM13A-AS1 on cervical cancer cell proliferation, apoptosis, invasion, and migration. A bioinformatics analysis and a dual-luciferase assay were carried to confirm the target relationship between FAM13A-AS1 or DDI2 and miRNA-205-3p. Finally, in vivo tumorigenesis experiments were performed in nude mice to explore the effect of FAM13A-AS1 expression on cervical cancer. Low FAM13A-AS1 expression and high miRNA-205-3p expression were observed in cervical cancer tissues and cell lines (SiHa and HeLa). Upregulating the expression of FAM13A-AS1 inhibited proliferation, migration, and invasion of SiHa and HeLa cells, while the apoptosis of SiHa and HeLa cells was increased. More importantly, LV-FAM13A-AS1 could improve tumor development in vivo. In addition, FAM13A-AS1 negatively regulated the expression of miRNA-205-3p, while miRNA-205-3p reduced DDI2 expression, and miRNA-205-3p mimic reversed the effects of FAM13A-AS1 overexpression in vitro. In conclusion, FAM13A-AS1 inhibits the progression of cervical cancer by targeting the miRNA-205-3p/DDI2 axis, suggesting that FAM13A-AS1 might be a potential target for cancer cell treatment.

## 1. Introduction

Cervical cancer is the second most common type of malignant tumor affecting women [1]. Recent data suggest that cervical cancer is caused by HPVs, smoking, genetics, early sexual life, and different kinds of environmental factors [2, 3]. Although traditional therapies including surgery, radiation therapy, targeted therapy, and chemotherapy are widely available for cervical cancer [4], treatment outcomes remain poor and most patients die after the onset of metastasis [3]. Therefore, it is urgent to clarify the molecular mechanisms underlying cervical cancer in order to identify targets for its treatment and to facilitate diagnosis and prognosis evaluation.

Long noncoding RNAs (lncRNAs) are a group of noncoding RNAs that exceed 200 nucleotides. lncRNAs are involved in modulating chromatin or acting as endogenous sponges of microRNAs that lead to their expression silence at the transcriptional level, thus leading to pathological changes [5]. Several lncRNAs have shown to play important roles in the promotion or suppression of cervical cancer by regulating various biological processes including cell cycle, survival, differentiation, and apoptosis [6, 7]. For instance, lncRNA C5orf66-AS1 negatively regulates the expression of miR-637 and promotes the invasion and growth of cervical cancer by adjusting RING1 levels [8]. Moreover, lncRNA-CTS was verified to be highly expressed in cervical cancer tissues and cells and was shown to promote cervical cancer progression by downregulating miR-505 expression and facilitating the endothelial-mesenchymal transition [9]. Li et al. revealed that lncRNA NCK1-AS1 accelerated the growth of cervical cancer by regulating the miR-6857/CDK1 axis [10]. Shao et al. demonstrated that lncRNA STXBP5-AS1 suppressed cervical cancer progression by targeting the miR-96-5p/PTEN axis [11]. Gong et al. showed that lncRNA HAND2-AS1 repressed cervical cancer progression by interaction with transcription factor E2F4 at the promoter of C16 or f74 lncRNA [12]. FAM13A-AS1 is a novel lncRNA discovered in thyroid cancer which shows potential for the prognosis of thyroid cancer [13]. However, its expression and mechanism in cervical cancer have not been elucidated.

Therefore, in the present study, we examined the function and mechanism of FAM13A-AS1 in cervical cancer. We confirm that FAM13A-AS1 upregulates DNA-damage inducible 1 homolog 2 (DDI2) expression through miRNA-205-3p to suppress cervical cancer progression, which implicates FAM13A-AS1 as a potential biomarker for cervical cancer treatments.

## 2. Materials and Methods

### 2.1. Patients and Samples

Tumor tissue samples from 30 cervical cancer patients were collected from the surgical specimen archives of Wuxi No. 2 People's Hospital between August 2019 and June 2020, with written informed consent from the patients. Inclusion criteria were as follows: patients diagnosed with direct operative squamous cell carcinoma at International Federation of Gynecology and Obstetrics (FIGO) stages I-II; clear preoperative biopsy and postoperative pathological diagnoses were available; this was the first cervical cancer diagnosis for patients, and no other chemotherapy or radiotherapy was administered before surgery. The animal experimental procedure was approved by the Ethics Committee of the Wuxi No. 2 People's Hospital.

### 2.2. Cell Lines and Cell Culture

Human cervical cell lines (HeLa cells and SiHa cells) and the human cervical epithelial cell line HUCEC were obtained from the Cell Bank of the Chinese Academy of Sciences (Shanghai, China). Cells in the third to eighth generation logarithmic growth phase were selected for testing. Cervical cancer cells were incubated in the Dulbecco's modified Eagle's medium (DMEM × high glucose; Gibco, Invitrogen) containing 10% fetal bovine serum (Gibco) at 37°C under 5% CO_2_ in a CO_2_ incubator.

### 2.3. Cell Transfection

In order to construct stable cells with FAM13A-AS1 knockdown, silencing RNA (siFAM13A-AS1) or negative control siRNA (siNC) was inserted into the pLKO.1 vector (Sigma). The target sequences of the siRNAs were as follows: siRNA1 sense: GCUGGAUUACAUAAUACUAUU, antisense: UAGUAUUAUGUAAUCCAGCUG; siRNA2 sense: GCAAGUUUGCUCUAUAGAAUA, antisense: UUCUAUAGAGCAAACUUGCUG; siRNA3 sense: GUAGAUGAGUGUUGUCUAAUG, antisense: UUAGACAACACUCAUCUACAG. In order to construct stable cells overexpressing FAM13A-AS1, the sequence of FAM13A-AS1 was subcloned into the pLV plasmid (Inovogen, KLV3501). TurboFect (Thermo) was used to cotransfect the lentiviral expression plasmid psPAX2 (Addgene) and pMD.2 G plasmid (Addgene) into 293T cells. After transfection, the lentiviral particles were harvested and used to infect SiHa or HeLa cells. After 48 h, puromycin (3 *μ*g/mL) was administered for 1 week to select stable cells.

### 2.4. RT-qPCR

The total RNA of cervical cancer tissues, SiHa, HeLa, and HUCEC cells were extracted by TRIzol reagent (Thermo Fisher) to detect the abundance of FAM13A-AS1, miRNA-205-3p, and DDI2 in cervical cancer tissues and cells. The TaqMan microRNA Reverse Transcription Kit or High Capacity cDNA Reverse Transcription Kit (Thermo Fisher) was used to reverse transcribe 1 *μ*g of RNA into cDNA. Quantitative PCR was carried out using SYBR Green Mix (Thermo Fisher) and a 7900HT Fast RT-qPCR machine (Thermo Fisher). The reaction conditions for qPCR were as follows: denaturation at 94°C for 15 s, annealing at 56°C for 30 s, and extension at 72°C for 60 s, 35 cycles. The primer sequences are given in Table 1. The relative levels of FAM13A-AS1, miRNA-205-3p, and DDI2 were calculated by the 2^−ΔΔC^tmethod. *β*-Actin was used for the internal control for FAM13A-AS1 and DDI2, and U6 was used for the internal control for miR-205-3p.

### 2.5. In Situ Hybridization (ISH) Assay

ISH analysis was performed to detect the expression level of FAM13A-AS1 in cervical cancer and normal tissues. The ISH probe was designed to target the following FAM13A-AS1 sequence: 5′-ATGCCTAACATATTATCTAGCCCT-3′. The tissue-fixed slides were digested with proteinase K (1 *μ*g/ml) at 37°C for 10 minutes, and then, the slides and probes were prehybridized at 42°C for 30 minutes at a final concentration of 1.5 *μ*g/ml. Incubate the slides in blocking buffer for 15 minutes and preabsorb with biotin-conjugated mouse antidigoxigenin (Boster, MK1003) at a dilution of 1 : 1000 at room temperature for 1 hour. Finally, hematoxylin was used as a background color, and 3,3′-diaminobenzidine was used to stain the slide to show the positively stained tissue area. Three biological replicates were performed.

### 2.6. Western Blotting

Protein was extracted from the experimental and control groups, electrophoretically separated by SDS-PAGE, and then transferred onto nitrocellulose membranes. The primary rabbit anti-human antibodies used for immunoblotting were as follows: *β*-actin (42 kDa, 1 : 1000, ab8226, Abcam) and DDI2 (45 kDa, 1 : 500, sc-514004, Santa Cruz). The samples were incubated overnight at 4°C and then washed with TBS-T three times. Goat anti-rabbit secondary antibody (1 : 5000, Biosharp, BL001 A) was incubated with the membrane for 1 h, followed by washing with TBS-T. The Odyssey infrared laser scanning imaging system (LI-COR Biosciences) was used to scan protein bands. Membrane bands were analyzed using Image *J* v1.48 software (National Institutes of Health, USA).

### 2.7. MTT Assay

HeLa cells in the logarithmic growth phase were collected, and 5 × 10^3^ cells were seeded in a 96-well culture plate with a cell culture solution containing 10% fetal bovine serum and DMEM. After culturing for 24 h, the supernatant was discarded and 20 *μ*l of sterile MTT (Sigma, M2128) was added to the wells, including three replicate wells for each time point. After 4 h of continuous culture, the supernatant was completely removed, DMSO at 150 *μ*l/well was added, and the wells were shaken for 10 min. The absorbance at a 492 nm wavelength was measured with a microplate reader, and the proliferation rate of each group was calculated.

### 2.8. Transwell Invasion and Migration Assay

The upper chamber of a transwell insert was spread with Matrigel for invasion assays (or left without Matrigel for migration experiments) one day before cells were inoculated in the upper chamber. A DMEM/high glucose medium with 10% FBS was added to the lower invasion chamber, and the cell suspension was transferred to the upper chamber at a density of 1 × 10^5^ cells/well. The cells were incubated for 24 h; then, a cotton swab was used to remove the Matrigel and the cells from the upper chamber. The cells that had migrated to the lower chamber were fixed with 4% paraformaldehyde and stained with 0.1% crystal violet. Photographs were captured using light microscopy (Olympus).

### 2.9. Flow Cytometry Analysis of Cell Apoptosis

Four transfected cell lines (LV-NC, LV-FAM13A-AS1, LV-siNC, and LV-siFAM13A-AS1) were collected and resuspended in binding buffer and used according to the instructions for the Annexin V-FITC/PI kit (Beyotime) for apoptosis detection. The cells were analyzed by flow cytometry, and the apoptotic rate was expressed as the percentage of cells in early apoptosis (Annexin V-FITC-positive and PI-negative) and late apoptosis (Annexin V-FITC-positive and PI-positive).

### 2.10. Dual-Luciferase Reporter Assay

The DIANA database (http://diana.imis.athena-innovation.gr/DianaTools/) and TargetScan (https://www.targetscan.org/vert_80/) were used to predict the binding sites for FAM13A-AS1 and DDI2 of miR-205-3p, respectively. The Shanghai Biotech Engineering Co., Ltd. provided the mutant and wild-type sequences for FAM13A-AS1 and DDI2. FAM13A-AS1 wild-type (WT), FAM13A-AS1 mutant (MUT), DDI2 WT, and DDI2 MUT reporter plasmids were transfected into 293 T cells with miRNA mimics and NC mimics. Lipofectamine 2000 was used for plasmid transfection, and Renilla luciferase plasmid was used for experimental control. Luciferase activity was measured 48 h after transfection using a dual-luciferase reporter gene assay system (Promega, Madison, USA).

### 2.11. In Vivo Nude Mouse Cervical Cancer Model

Nude mice (4–8 weeks old) were purchased from the Experimental Animal Center of Yangzhou University. After the nude mice were reared for one week, the same volume of control, LV-NC, or LV-FAM13A-AS1 cell suspensions were injected subcutaneously (0.5 mL, 2 × 10^6^ mL^−1^ cells). There were six nude mice in each group. The tumor growth of the mice in each group was monitored every 7 days. After 35 days, the tumor mass in the nude mice was dissected.

### 2.12. Immunohistochemical Assay

The tissues were fixed in a formalin solution overnight, dehydrated in ethanol, embedded in paraffin, and sectioned at 5 *µ*m. The slides were blocked with 5% normal goat serum and incubated with anti-Ki67 (1 : 500) and anti-DDI2 (1 : 100) at 4°C. After washing with PBS, the slides were incubated with goat anti-rabbit horseradish peroxidase (Vector Laboratories, USA) at room temperature for 30 min. A DAB kit (DAB-1031, MXB Biotechnologies, China) was used to visualize the immunohistochemical reactions. Photographs were captured using light microscopy (Olympus Ckx53, Japan).

### 2.13. Statistical Analysis

Quantitative data are presented as means ± standard deviation. SPSS software (version 19.0; IBM, USA) and GraphPad Prism 6.0 software (GraphPad Software Inc., USA) were used. The *t*-test and one-way ANOVA were performed, followed by Student–Newman–Keuls tests to determine statistical differences. Three biological replicates were performed. *P* < 0.05 was considered to be a significant difference.

## 3. Results

### 3.1. FAM13A-AS1 Was Weakly Expressed in Cervical Cancer

**Figure 1:**
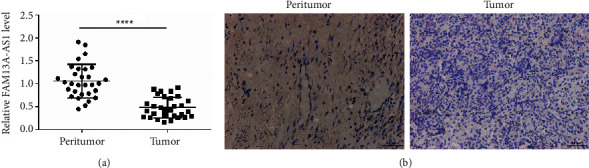
FAM13A-AS1 is weakly expressed in cervical cancer. (a) RT-qPCR was used to detect the expression of FAM13A-AS1 in tumor tissues and normal tissues. (b) ISH assay performed to detect the expression of FAM13A-AS1 using a specific probe.

ISH assays and RT-qPCR were performed to detect the expression of FAM13A-AS1 using a specific probe. The expression of FAM13A-AS1 was significantly lower in cervical cancer tissues compared with the normal group (Figures 1(a) and 1(b)).

### 3.2. FAM13A-AS1 Inhibited Proliferation and Promoted Apoptosis of Cervical Cancer Cells

**Figure 2:**
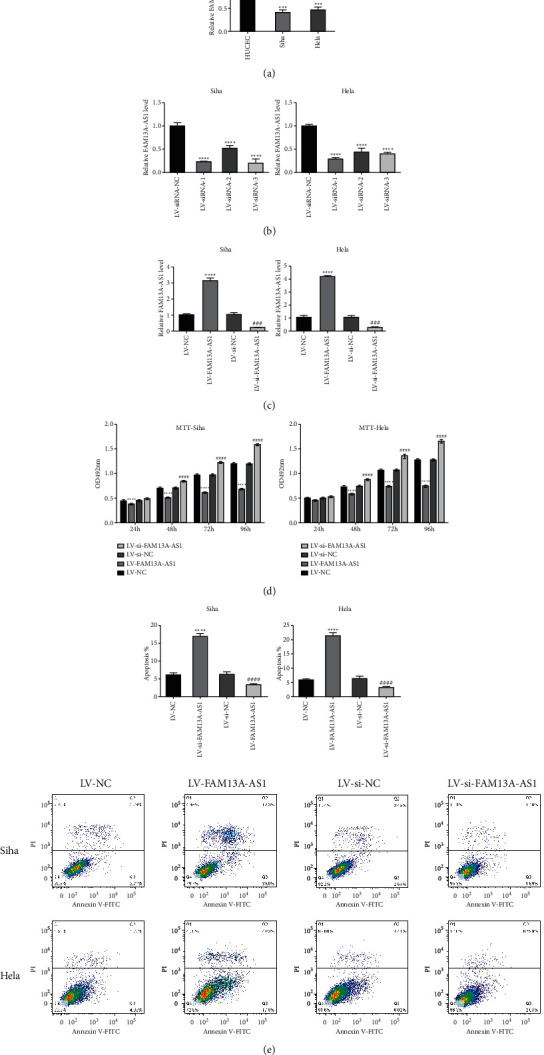
Effects of FAM13A-AS1 on proliferation and apoptosis of cervical cancer cells. (a) Expression of FAM13A-AS1 in cervical cancer cell lines detected by RT-qPCR. (b) SiHa and HeLa cells transfected with LV-siNC (negative control, NC) or LV-siFAM13A-AS1. FAM13A-AS1 levels were examined by RT-qPCR. (c) SiHa and HeLa cells transfected with LV-FAM13A-AS1 or LV-vector (NC). FAM13A-AS1 levels were examined by RT-qPCR. (d) Cell proliferation detected by the MTT kit. (e) Flow cytometry assays used to detect cell apoptosis. ^*∗*^*P* < 0.05, ^*∗∗∗*^*P* < 0.001, ^*∗∗∗∗*^*P* < 0.0001.

In order to examine the effect of FAM13A-AS1 on the proliferation and apoptosis of cervical cancer cells, RT-qPCR was used to evaluate the expression of FAM13A-AS1 in SiHa and HeLa cells. The results in Figure 2(a) shows that the expression of FAM13A-AS1 in SiHa and HeLa cells was significantly lower than that in HUCEC cells. The FAM13A-AS1 plasmid was transfected into SiHa and HeLa cells, and transfection efficiency was checked by RT-qPCR. The results showed that FAM13A-AS1 expression significantly decreased in LV-siFAM13A-AS1 stable cell lines while increased in LV-FAM13A-AS1 stable cell lines compared with the control group (Figures 2(b)–2(e)). Cell viability was detected by the MTT assay, and the results showed that viability in the LV-FAM13A-AS1 group was lower than in the LV-siFAM13A-AS1 group and control group (Figure 2(d)). In addition, a flow cytometry assay showed that downregulation of FAM13A-AS1 notably inhibited apoptosis in SiHa and HeLa cells, while compared with the control group, overexpression of FAM13A-AS1 promoted the apoptotic process (Figure 2(e)).

### 3.3. FAM13A-AS1 Inhibited Invasion and Migration of Cervical Cancer Cells

**Figure 3:**
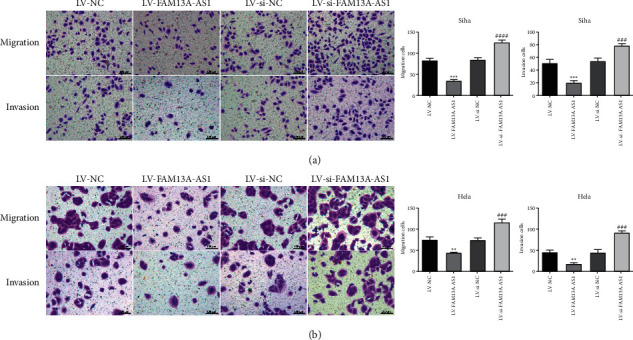
Effects of FAM13A-AS1 on migration and invasion of cervical cancer cells. (a, b) Transwell chamber assays analyzed the effect of FAM13A-AS1 on cervical cancer cell migration and invasion. Statistical analysis shows the effect of FAM13A-AS1 on migration and invasion of cervical cancer cells, compared with the NC group. ^*∗∗*^*P* < 0.01, ^*∗∗∗*^*P* < 0.001, ^*∗∗∗∗*^*P* < 0.0001.

Transwell assays were used to examine the effects of FAM13A-AS1 on the invasion and migration of SiHa and HeLa cells. The results showed that compared with the control group and LV-NC group, the number of invading and migrating cells in the LV-FAM13A-AS1 group was significantly decreased but increased in the LV-siFAM13A-AS1 group (Figures 3(a) and 3(b)). The results indicated that FAM13A-AS1 inhibited the invasion and migration of cervical cancer cells.

### 3.4. FAM13A-AS1 Was a Sponge of miRNA-205-3p

**Figure 4:**
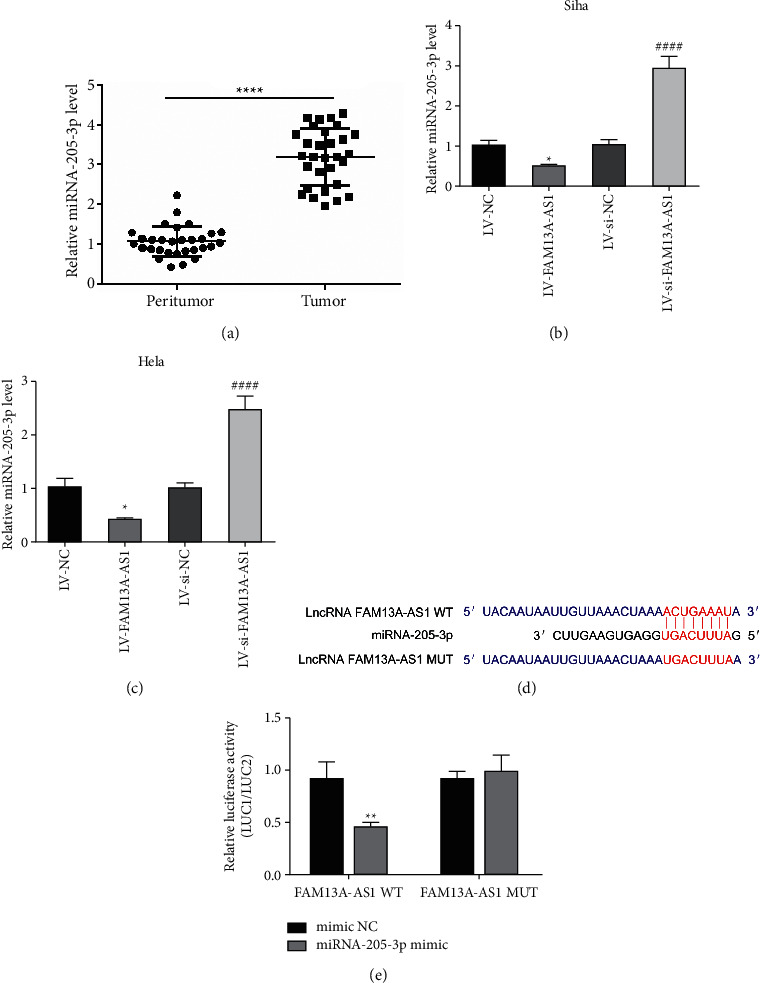
FAM13A-AS1 was a sponge of miRNA-205-3p. (a) miR-205-3p expression in cervical cancer tissues detected by RT-qPCR. (b, c) miR-205-3p expression in cervical cancer cell lines (SiHa and HeLa) detected by RT-qPCR. (c) The binding sites of FAM13A-AS1 on miR-205-3p predicted by DIANA tools. (d) The interaction between FAM13A-AS1 and miR-205-3p determined by dual-luciferase reporter assay. ^*∗*^*P* < 0.05, ^*∗∗*^*P* < 0.01, ^*∗∗∗∗*^*P* < 0.0001.

RT-qPCR was performed to detect the expression of miR-205-3p in cervical cancer tissue and cell lines. The results showed that the expression of miR-205-3p in cervical cancer tissue was significantly higher than in the control group (Figure 4(a)), and a negative correlation was found between FAM13A-AS1 and miR-205-3p in SiHa and HeLa cells (Figures 4(b) and 4(c)). A dual-luciferase reporter assay was performed to analyze the targeting of miR-205-3p to FAM13A-AS1 in cervical cancer by establishing FAM13A-AS1 constructs with WT and Mut binding sites for miR-205-3p. Decreased FAM13A-AS1 luciferase activity was observed after transfection of miR-205-3p (Figures 4(d) and 4(e)), suggesting that FAM13A-AS1 was a sponge of miR-205-3p.

### 3.5. DDI2 Was the Target of miRNA-205-3p

**Figure 5:**
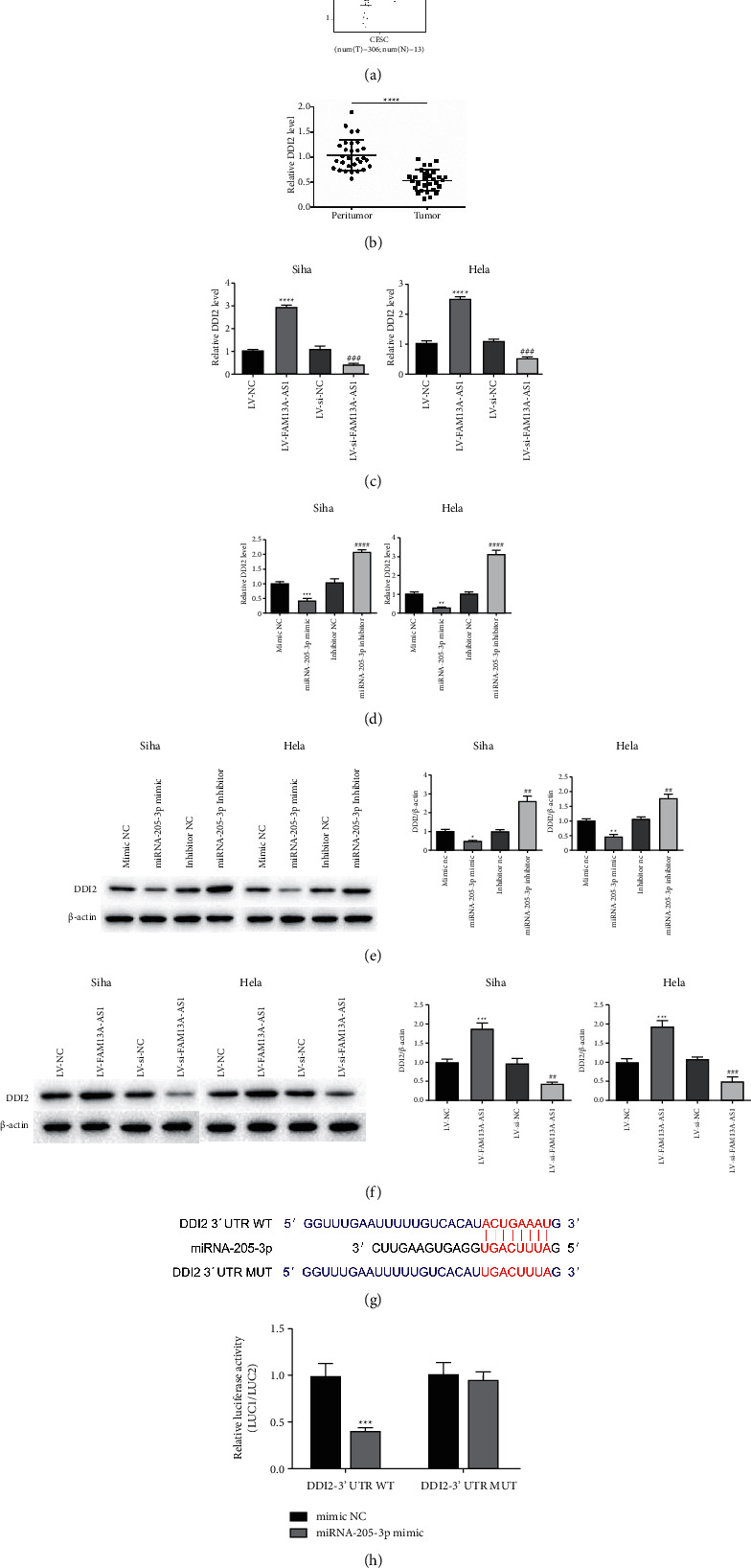
miR-205-3p interacts with DDI2. (a) The expression of DDI2 in cervical cancer patients from TCGA performed by gene expression profiling interactive analysis. (b) RT-qPCR was used to detect the expression of DDI2. (c), (d) Relative DDI2 expression after transfection of FAM13A-AS1, miR-205-3p inhibitor, or miR-205-3p mimic. (e), (f) Western blot assay detected the protein level of DDI2 after transfection. (g) The binding site of miR-205-3p and DDI2. (h) Luciferase reporter experiments confirmed that miR-205-3p targeted DDI2. ^*∗*^*P* < 0.05, ^*∗∗*^*P* < 0.01, ^*∗∗∗*^*P* < 0.001, ^*∗∗∗∗*^*P* < 0.0001.

In order to know the targets of miR-205-3p, TargetScan was used to predict the targets of miR-205-3p. We further found that the expression of DDI2 was lower in the tumor group compared with the normal group of the targets with a high binding score (Figure 5(a)). RT-qPCR also confirmed that the expression of DDI2 in cervical cancer was lower than in normal tissue (Figure 5(b)). In cervical cancer cell lines, overexpression of FAM13A-AS1 increased the DDI2 expression level (Figure 5(c)), and the miR-205-3p inhibitor also increased the expression of DDI2, while miR-205-3p mimic reversed these effects (Figure 5(d)). Similar results were obtained by Western blot (Figures 5(e) and 5(f)). According to the DIANA website, miR-205-3p targets DDI2, and the binding site is shown in Figure 5(g). The results of luciferase reporter experiments also indicated that miR-205-3p targets DDI2 (Figure 5(h)).

### 3.6. LV-FAM13A-AS1 Inhibited Development of Tumors In Vivo

**Figure 6:**
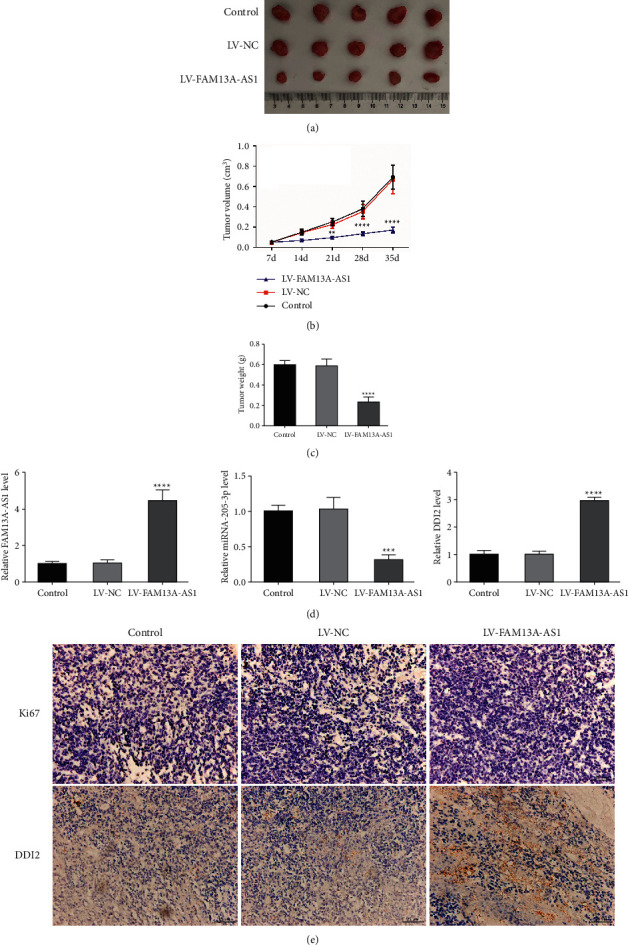
LV-FAM13A-AS1 inhibited development of tumors in vivo. (a) HeLa cells (2  ×  10^6^ cells per mouse, *n* = 6) inoculated in NOD/SCID nude mice to establish subcutaneous xenograft tumors. The mice were euthanatized at 35 days and the tumors were dissected. (b), (c) Relative tumor volumes and weights measured at 35 days. (d) RT-qPCR was used to detect the expression of FAM13A-AS1, miR-205-3p, and DDI2 in tumor tissues. (e) Representative images of immunohistochemical staining for Ki67 and DDI2 in different groups. ^*∗∗*^*P* < 0.01, ^*∗∗∗∗*^*P* < 0.0001.

Tumor volumes and weights in mice inoculated with LV-FAM13A-AS1 cells were reduced compared with the control and LV-NC group after transfection (Figures 6(a)–6(c)). The expression of FAM13A-AS1, miR-205-3p, and DDI2 in tumor tissues was evaluated by RT-qPCR. Results suggested that the expression of FAM13A-AS1 and DDI2 increased in the LV-FAM13A-AS1 group, while miR-205-3p expression was decreased (Figure 6(d)). Immunohistochemistry was performed to detect the expression of DDI2 and the tumor cell proliferation factor Ki67. Results showed that the expression of DDI2 was increased, while the expression of Ki67 was decreased in the LV-FAM13A-AS1 group compared with the NC group (Figure 6(e)).

## 4. Discussion

Cervical cancer is one of the most common malignant tumors worldwide. Human papillomavirus (HPV) infection is an important cause of cervical cancer [14, 15]. Persistent HPV infection may be related to cervical intraepithelial neoplasia of different grades and invasive cancer [16]. Radiotherapy and chemotherapy are the main therapies for the treatment of cervical cancer [17]. However, the resistance of cervical cancer cells to therapeutic drugs and the side effects of chemotherapy are the main obstacles to the treatment of cervical cancer [18]. Therefore, the discovery of new biomarkers for cervical cancer is necessary for the early diagnosis, prevention, and treatment [19].

lncRNAs are a class of noncoding RNA containing more than 200 nucleotides that were initially considered transcriptional noise [20]. Previous studies have reported that lncRNAs have functions in numerous biological activities, including cell cycle regulation [21], stem cell differentiation, the immune response [22], cancer progression [23], and chemotherapy resistance [24]. Zhang et al. [25] suggested that abnormal expression of lncRNAs plays an important role in tumorigenesis, invasion, and metastasis, including in cervical cancer. FAM13A-AS1 has been reported as a novel genetic locus in clinical thyroid disease and bladder cancer, which can inhibit tumor growth and prolong the survival time of patients [13, 26]. Recently, Wang et al. demonstrated that FAM13A-AS1 promoted renal carcinoma tumorigenesis through sponging miR-141-3p to upregulate NEK6 expression, suggesting that FAM13A-AS1 played an important role in the progression of cervical cancer [27]. However, the functions of FAM13A-AS1 in cervical cancer have not been studied. The current study was designed to investigate the roles of FAM13A-AS1 in cervical cancer and the underlying mechanisms of cancer development and progression. In the present study, we found that the expression levels of FAM13A-AS1 were revealed to be significantly downregulated in cervical cancer tissues and were related to the poor survival of cervical cancer patients. In addition, we found that overexpression of FAM13A-AS1 inhibited proliferation, migration, and invasion of cervical cancer cells in vitro and blocked tumor growth in vivo. Therefore, FAM13A-AS1 is likely to act as a tumor suppressor in cervical cancer.

A number of studies have shown that in cervical cancer, lncRNAs competitively decoy miRNAs via miRNA response elements (MREs), and this process reduces the binding of miRNAs to their target mRNAs, which indirectly upregulates the level of downstream mRNAs [28–30]. Thus, we assumed that FAM13A-AS1 might function as a molecular sponge in suppressing cervical cancer. It has been shown that miR-205-3p is highly expressed in cervical cancer patients and is associated with poor survival [31]. Our results indicate that the expression of miR-205-3p is significantly downregulated in cervical cancer tissues and cells. FAM13A-AS1 and miR-205-3p were negatively correlated in cervical cancer. With the help of the DIANA database, we found that miR-205-3p has a potential binding site targeting FAM13A-AS1. The dual-luciferase reporter gene assay confirmed that FAM13A-AS can bind with miR-205-3p to inhibit its level, suggesting that FAM13A-AS could act as a sponge to bind with miR-205-3p.

DDI2 is a DNA damage-inducing protein homolog. The functions of DDI2 in cancer biology have only been reported in colorectal cancer and thyroid cancer [32, 33]. In the present study, we indicated that the expression of DDI2 in cervical cancer tissue was significantly lower than in normal tissue. Dual-luciferase reporter gene assays showed that DDI2 is a direct target of miR-205-3p. Further analysis showed that the expression of DDI2 could be regulated by miR-205-3p both in vitro and in vivo.

In conclusion, FAM13A-AS inhibited the progression of cervical cancer by targeting the miR-205-3p/DDI2 axis. These findings suggest that FAM13A-AS might be a new target for cervical cancer treatment.

## Figures and Tables

**Table 1:** Primer sequences used in real-time PCR.

Primer name	Primer sequence
FAM13A-AS1	Forward (F): 5′-CAAATATGGGTAAGGAGG-3′ Reverse (R) 5′-GTTTAGAACTATGAGGGACT-3′
miRNA-205-3p	Forward (F): 5′-GGCAGGGATTTCAGTGGAG-3′
DDI2	Forward (F): 5′'-TTCCCAAACTTACCCCGAATAGA-3′ Reverse (R): 5′-GGAGCAAGGCTGGATTGTC-3′
*β*-Actin	Forward (F): 5′-TTCCCAAACTTACCCCGAATAGA-3′ Reverse (R): 5′-GGGCACGAAGGCTCATCATT-3′
U6	Forward (F): 5′-CTCGCTTCGGCAGCACA-3′ Reverse (R): 5′-AACGCTTCACGAATTTGCGT-3′

## Data Availability

The data used to support the findings of this study are included within the article.

## References

[1] Rahimi S., Marani C., Gardner F., Yeoh C. C., Akaev I., Votano S. (2018). Endocervicoscopy and biopsy to detect cervical intraepithelial squamous neoplasia in nonvisible squamocolumnar junction with unsatisfactory colposcopy: a pilot study. *Technology in Cancer Research and Treatment*.

[2] Silva G., Nunes R., Morale M., Boccardo E., Aguayo F., Termini L. (2018). Oxidative stress: therapeutic approaches for cervical cancer treatment. *Clinics*.

[3] Gauri A., Messiah S. E., Bouzoubaa L. A., Moore K. J., Koru-Sengul T. (2018). Cervical cancer sociodemographic and diagnostic disparities in Florida: a population-based study (1981-2013) by stage at presentation. *Ethnicity and Health*.

[4] Diao W., Guo Q., Zhu C., Song Y., Chen H. (2020). USP18 promotes cell proliferation and suppressed apoptosis in cervical cancer cells via activating AKT signaling pathway. *BMC Cancer*.

[5] Bo N., Hao Ya-F., He X.-Q., Li K., Wang R.-L., Wang (2017). The role of miRNA and lncRNA in gastric cancer. *Oncotarget*.

[6] Sun W., Shen N. M., Fu S. L. (2019). Involvement of lncRNA-mediated signaling pathway in the development of cervical cancer. *European Review for Medical and Pharmacological Sciences*.

[7] He J., Huang B., Zhang K., Liu M., Xu T. (2020). Long non-coding RNA in cervical cancer: from biology to therapeutic opportunity. *Biomedicine & Pharmacotherapy*.

[8] Rui X., Xu Y., Jiang X., Ye W., Huang Y., Jiang J. (2018). Long non-coding RNA C5orf66-AS1 promotes cell proliferation in cervical cancer by targeting miR-637/RING1 axis. *Cell Death & Disease*.

[9] Feng S., Liu W., Bai X., Pan W., Tan W. (2019). LncRNA-CTS promotes metastasis and epithelial-to-mesenchymal transition through regulating miR-505/ZEB2 axis in cervical cancer. *Cancer Letters*.

[10] Li H., Jia Y., Cheng J., Liu G., Song F. (2018). LncRNA NCK1-AS1 promotes proliferation and induces cell cycle progression by crosstalk NCK1-AS1/miR-6857/CDK1 pathway. *Cell Death & Disease*.

[11] Shao S., Wang C., Wang S., Zhang H., Zhang Y. (2019). LncRNA STXBP5-AS1 suppressed cervical cancer progression via targeting miR-96-5p/PTEN axis. *Biomedicine & Pharmacotherapy*.

[12] Gong J., Fan H., Deng J., Zhang Q. (2020). LncRNA HAND2-AS1 represses cervical cancer progression by interaction with transcription factor E2F4 at the promoter of C16orf74. *Journal of Cellular and Molecular Medicine*.

[13] Rao Y., Liu H., Yan X., Wang J. (2020). Silico analysis identifies differently expressed lncRNAs as novel biomarkers for the prognosis of thyroid cancer. *Computational and Mathematical Methods in Medicine*.

[14] Woodman C. B. J., Collins S. I., Young L. S. (2007). The natural history of cervical HPV infection: unresolved issues. *Nature Reviews Cancer*.

[15] Cecilia R. A., Mark S., Rolando H. (2008). Rapid clearance of human papillomavirus and implications for clinical focus on persistent infections. *Journal of the National Cancer Institute*.

[16] Smith R. A., Andrews K. S., Brooks D. (2015). *Cancer Screening in the United States, 2015: A Review of Current American Cancer Society Guidelines and Current Issues in Cancer Screening*.

[17] Lee K. B., Shim S. H., Lee J. M. (2018). Comparison between adjuvant chemotherapy and adjuvant radiotherapy/chemoradiotherapy after radical surgery in patients with cervical cancer: a meta-analysis. *Journal of Gynecologic Oncology*.

[18] Haie-Meder C., Morice P., Castiglione M. (2012). Cervical cancer: ESMO Clinical Practice Guidelines for diagnosis, treatment and follow-up. *Annals of Oncology Official Journal of the European Society for Medical Oncology*.

[19] Ji Y. Y., Meng M., Miao Y. (2020). lncRNA SNHG1 promotes progression of cervical cancer through miR-195/NEK2 Axis. *Cancer Management and Research*.

[20] Deniz E., Erman B. (2017). Long noncoding RNA (lincRNA), a new paradigm in gene expression control. *Functional & Integrative Genomics*.

[21] Hu Y. W., Kang C. M., Zhao J. J. (2017). LncRNA PLAC2 down‐regulates RPL36 expression and blocks cell cycle progression in glioma through a mechanism involving STAT1. *Journal of Cellular and Molecular Medicine*.

[22] Liu H., Zhang L., Ding X., Sui X. (2021). LINC00861 inhibits the progression of cervical cancer cells by functioning as a ceRNA for miR513b5p and regulating the PTEN/AKT/mTOR signaling pathway. *Molecular Medicine Reports*.

[23] Wang M., Zhou L., Yu F., Zhang Y., Li P., Wang K. (2019). The functional roles of exosomal long non-coding RNAs in cancer. *Cellular and Molecular Life ences*.

[24] Zhang F., Zhang L., Zhang C. (2015). Long noncoding RNAs and tumorigenesis: genetic associations, molecular mechanisms, and therapeutic strategies. *Tumour Biology the Journal of the International Society for Oncodevelopmental Biology & Medicine*.

[25] Zhang J., Hu S. L., Qiao C. H. (2018). *LncRNA-NEF Inhibits Proliferation, Migration and Invasion of Esophageal Squamous-Cell Carcinoma Cells by Inactivating Wnt/β-Catenin Pathway*.

[26] Sun Z., Jing C., Xiao C., Li T. (2020). An autophagy-related long non-coding RNA prognostic signature accurately predicts survival outcomes in bladder urothelial carcinoma patients. *Aging (Albany NY)*.

[27] Wang X. J., Li S., Fang J., Yan Z. J., Luo G. C. (2022). LncRNA fam13a-AS1 promotes renal carcinoma tumorigenesis through sponging miR-141-3p to upregulate NEK6 expression. *Frontiers in Molecular Biosciences*.

[28] Luan X., Wang Y. (2018). LncRNA XLOC_006390 facilitates cervical cancer tumorigenesis and metastasis as a ceRNA against miR-331-3p and miR-338-3p. *J Gynecol Oncol*.

[29] Xia M., Duan L. J., Lu B. N., Pang Y. Z., Pang Z. R. (2021). LncRNA AFAP1-AS1/miR-27b-3p/VEGF-C axis modulates stemness characteristics in cervical cancer cells. *Chinese Medical Journal*.

[30] Zhao H., Hu G. M., Wang W. L., Wang Z. H., Fang Y., Liu Y. L. (2019). LncRNA TDRG1 functions as an oncogene in cervical cancer through sponging miR-330-5p to modulate ELK1 expression. *European Review for Medical and Pharmacological Sciences*.

[31] Wang Y., Zhang Z., Tao P., Reyila M., Yang J. (2020). The abnormal expression of miR-205-5p, miR-195-5p, and VEGF-A in human cervical cancer is related to the treatment of venous thromboembolism. *BioMed Research International*.

[32] Tomei S., Marchetti I., Zavaglia K. (2012). A molecular computational model improves the preoperative diagnosis of thyroid nodules. *BMC Cancer*.

[33] Lei L., Zhao X., Liu S., Cao Q., Yan B., Yang J. (2019). MicroRNA-3607 inhibits the tumorigenesis of colorectal cancer by targeting DDI2 and regulating the DNA damage repair pathway. *Apoptosis*.

